# Case report of open appendectomy in treating acute perforated appendicitis with necrotizing fasciitis of the abdominal wall: A rare complication of a common disease

**DOI:** 10.1002/ccr3.5354

**Published:** 2022-02-03

**Authors:** Mohsen Rakhsha, Rezvan Hosseinzadeh, Dariush Hosseinzadeh, Morteza Behnamfar, Kataneh Kazemi

**Affiliations:** ^1^ 196469 Department of Surgery School of Medicine North Khorasan University of Medical Sciences Bojnurd Iran; ^2^ Babol University of Medical Sciences Babol Iran; ^3^ Bogomolets National Medical University Kyiv Kyiv Ukraine; ^4^ 196469 School of Medicine North Khorasan University of Medical Sciences Bojnurd Iran

**Keywords:** acute appendicitis, case report, necrotizing fasciitis, perforation

## Abstract

Acute appendicitis can be associated with uncommon complications such as necrotizing fasciitis. We present a case of a 37‐year‐old woman referred to our hospital with a 1‐week history of significant weakness, anorexia, and mild abdominal pain. According to laboratory and radiographic data, the patient was diagnosed with perforated appendicitis and gangrene.

## Graphical abstract

If you experience sudden abdominal pain, please see your doctor before attempting any self‐medication to prevent your condition from getting worse.

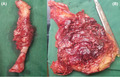

## 1 INTRODUCTION

Acute appendicitis is one of the most common emergent surgeries. Generally, appendicitis is inflammation of the appendix,^1^ and appendicitis occurs primarily in the second and third decades of life, but its incidence increases in the elderly. Intense an infected appendix is by all accounts the aftereffect of an essential deterrent of the supplement.^2^ Symptoms commonly totaling together occur right lower abdominal tormented sensation, nausea, vomiting, and decreased appetite.^1^ CT examination has been demonstrated to be more exact than ultrasound in recognizing intense an infected appendix.^3^ The rate of postoperative complications of appendix resection, such as intra‐abdominal abscess and wound infection, is about 8 to 13%.^4^ The mortality rate is estimated to be less than 1.3%, but if delayed, it can be up to 6 times higher and can cause severe complications.^5^ Almost 7% of people may develop appendicitis at least once in their lives. Clinical signs assigned to appendicitis diagnosis include fever, anorexia, right lower quadrant (RLQ) pain, and elevated white blood cell count (WBC) that can vary in individuals and cause a delayed diagnosis and treatment.^6^

Pregnant women (43%) are more likely to develop perforated appendicitis than others (15%). During pregnancy, especially in the second trimester, appendicitis can occur with various clinical manifestations, causing many diagnostic problems.^7^ When managing such patients, the clinical concern is whether to prescribe antibiotics or perform emergency surgery.^8^ Although wide‐spectrum antibiotic treatment is thought to be a safe management plan in treating appendicitis, it has a 7% failure rate, most commonly seen in immunosuppressed patients who mainly use corticosteroids.^9^

Necrotizing fasciitis (NF) causes a series of deadly infections that include inflammation followed by necrosis of the skin, subcutaneous fat, and fascia, which may also be associated with inflammation of the underlying muscle and may even lead to systemic sepsis.^10^, ^11^ It can occur with redness, swelling, and pain.^12^

Abdominal wall NF is a lethal and rare complication of acute appendicitis. The disease is mainly observed in immunodeficient patients, in whom the mortality rate can be up to 30%.^4^, ^8^, ^9^ Adult NF occurs more frequently in the abdomen, perineum, and limbs, but in children, especially infants, it usually occurs in the trunk.^13^ NF in infants after acute appendicitis is rare and fatal, but exceptions have also been reported, which require prompt diagnosis with aggressive medical and surgical interventions. Delay in diagnosis or treatment could lead to irreversible complications and even death. Therefore, emergency surgery should be performed on these patients immediately after an accurate and evident diagnosis.^11^

In this report, we present a perforated appendicitis case that caused NF of the abdominal wall.

## 2 CASE PRESENTATION

A 37‐year‐old woman with a history of mental retardation and schizophrenia was admitted to Imam Hassan Hospital, Bojnurd, Iran. Her symptoms began 7 days ago with severe and progressive weakness, anorexia, and mild abdominal pain. Body temperature was 37.8°C, blood pressure was 95/60 mmHg, heart rate was 110 beats/min, and respiratory rate was 18 breaths/min. On physical examination, there was a 10 × 10 mass with subcutaneous emphysema in the RLQ of her abdomen.

The patient's history was as follows: dry mucosa in the oropharyngeal cavity, edema, especially in the finger areas, neck pain, shortness of breath, anorexia, nausea, constipation, urinary incontinence, anxiety disorders, psychosis (exacerbated after her first delivery at age 25), paraparesis, tremor, anemia, dizziness, nasal and sinus bleeding, persistent nasal discharge, dry skin in the lower extremity areas, weight loss, and fever. A family history of stroke, mitral regurgitation, kidney stones, hyperlipidemia, and diabetes mellitus. The patient's drug history included olanzapine, propranolol, valproic acid, and fluoxetine. Her allergy history was not remarkable.

Obtained laboratory and paraclinical data showed 12800 white blood cells with 74% neutrophils, 31% hematocrit, and 0.6 mg/dl creatinine. Spiral abdominal and pelvic computed tomography scan (CT scan) with contrast showed the liver, pancreas, and spleen that were normal in size and density without space‐occupying lesions (SOLs), but dilation of the bile ducts inside and outside the liver was observed. There was slight hydronephrosis in the right kidney. The heterogeneous multi‐locus center image with peripheral enhancement and liquid density of the internal mixture with 107 × 81 mm dimensions was observed in the RLQ region. Several gas bubbles were visible on the non‐dependent surface of the necrotic section. Evidence of fat‐stranding inflammation in the surrounding adipose tissue and the mass effect on the adjacent loops of the small intestine was also observed with the thickening of the wall of the mentioned loops. The possibility of complicated appendicitis with phlegmon formation and the early‐stage abscess accumulation was raised. Ascites and lymphadenopathy were not observed. In the visible parts of the lungs, pleural effusion was observed. The bladder was normal, and there was no obvious pathology in the adnexa.

The following was observed in a complete abdominal and pelvic ultrasonography: The spleen was normal in shape, size, and parenchymal echogenicity. An increase in liver parenchymal echogenicity was seen due to a grade two fatty liver. There was no bile duct ectasia in the liver, and port and CBD were normal. The gallbladder was completely clothed. The pancreas, aorta, para‐aorta, and kidneys were normal. The left ovary and uterus could not be examined, but the right ovary was normal in shape, size, and stromal echogenicity. The image of the heterogeneous region in the RLQ with a phlegmon view was observed with dimensions of 60 × 95 mm and a mass effect among intestinal loops. Mild free fluid was found in the Morrison pouch.

Based on the acquired data, the differential diagnosis included gastroenteritis, Meckel's diverticulum, cholecystitis, ectopic pregnancy, pancreatitis, and appendicitis. According to laboratory data, CT scan and ultrasound were done. The patient was diagnosed with perforated appendicitis and gangrene, with the tip of the appendix turned into NF in the RLQ wall.

**FIGURE 1:**
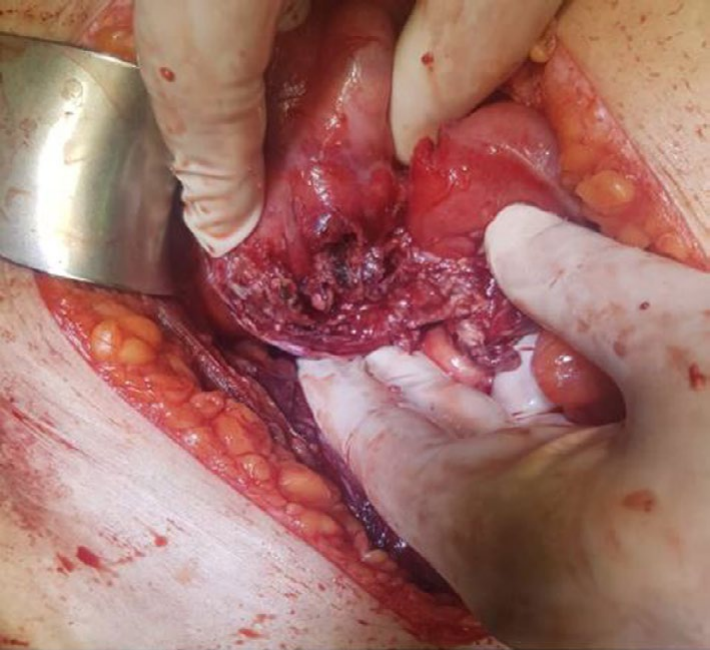
Surgical view of the resected appendix

**FIGURE 2:**
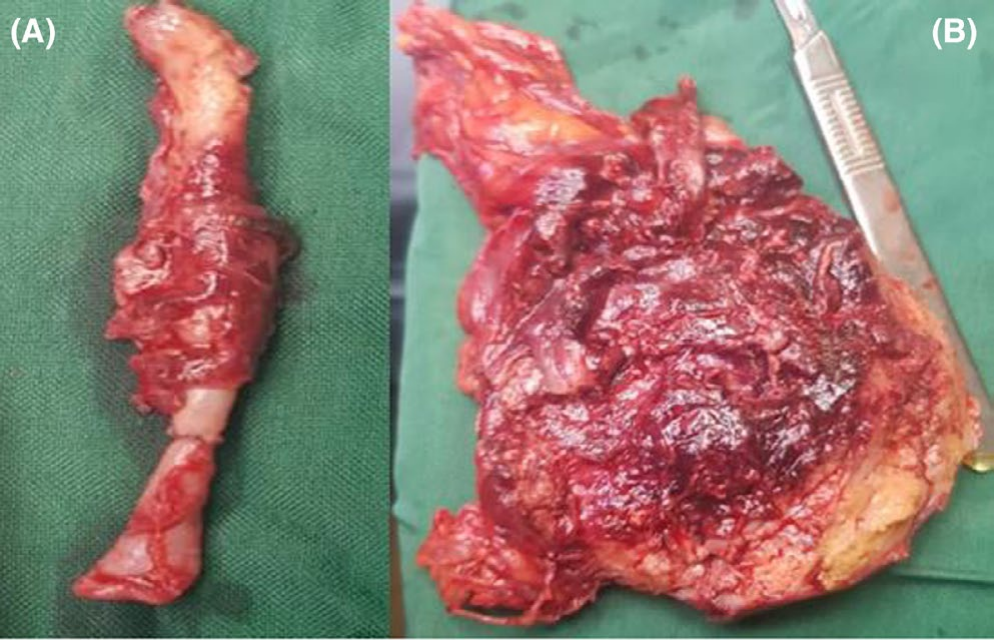
(A) Appendix, (B) Fibrofatty tissue with severe inflammation, granulation tissue, and necrosis

On the third day, an internal medicine consultation suggested that the patient be transferred to the operating room for an open appendectomy (Figure 1.). The patient was a candidate for daily debridement after the procedure, which continued for 4 days (Figure 2.). The patient underwent general anesthesia on the first day, and a Rocky–Davis incision was performed. The abdomen was rinsed daily, a long gauze covered the open wall. On the fourth day, the patient was implanted with a drain, and a secondary abdominal repair procedure was performed for the patient.

The resected tissue was sent for pathological examinations, and no malignancy was observed. Despite the medical staff's recommendations, the patient was not transferred to the psychiatric ward and was discharged with her and her family's consent. At the 1‐month follow‐up, the wound was almost completely healed, and no more complaints happened for the patient.

## 3 DISCUSSION

Our study observed 16 reports of patients with NF caused by appendicitis published in English literature and cited in Pub Med. Nine of these patients were male, and seven were female, aged 19 days to 76 years (mean age: 45 years). Six (37.5%) patients died, indicating a high mortality rate.

Necrotizing fasciitis (NF) is a soft tissue infection that leads to progressive necrosis in fascia and subcutaneous tissues. As the disease progresses, the blood vessels that nourish the skin become thrombotic, causing the skin to thicken and form gangrenous blisters. Also, skin color can be used to indicate the disease's progression. At first, the skin color was normal, but after 36 h, it changed to red and purple and finally turned to blue‐gray.^6^ The risk of NF is higher in patients with immune deficiency and underlying diseases such as liver disease, diabetes, malignancy, and kidney disease. In healthy people, it can be caused by any surgery or wound. NF has two types. Most cases are of the first type and are multi‐microbial in nature, with a mixture of aerobic and anaerobic bacterial species. The second type comprises a single type of bacterial infection, usually *Staphylococcus aureus* or *Streptococcus*.^13^, ^14^ General symptoms of NF that have been reported in various studies include fever, tenderness, pain, rashes, and erythema. NF can also be associated with non‐specific symptoms, including cough, sore throat, headache, abdominal pain, confusion, chills, syncope, diffuse myalgia, vomiting, diarrhea, and shortness of breath.^13^ NF can be a severe complication of several diseases, including perforated viscus, appendicitis, renal calculi after abdominal injury, a colo‐cutaneous fistula, postoperative complications, and incarcerated hernia.^15^

Perforated appendicitis that leads to necrosis of the abdominal wall is rare and fatal. The appendix is pierced through the abdominal wall, causing a progressive bacterial infection in the abdominal cavity.^5^ Appendicitis with NF is rare in neonates but is more commonly associated with perforation. NF in infants is usually associated with another underlying disease, such as mastitis, trauma, necrotizing enterocolitis, balanitis, bullous impetigo, immunodeficiency, omphalitis, or surgical wound infection.^11^

C‐reactive protein (CRP) is an important finding early in the disease course. Other possible indicators include increased white blood cell count, reduced hemoglobin levels, increased blood sugar, decreased sodium ion level, and raised creatinine. Furthermore, it is essential to remember that the data obtained from paraclinical and laboratory tests are somehow non‐specific and always should accompany a detailed and accurate medical history and proper physical examinations.^14^ In the first stage, the diagnosis of NF is based on clinical and physical examination. The patients complain of severe pain with the characteristic examination features that include edema and tenderness extending beyond the limits of cutaneous erythema, crepitus, and skin vesicles. The results of the laboratory exams show a polymorphonuclear leukocytosis. Due to hemolytic bacteria's effect on red blood cells, the laboratory reports showed anemia and hyperbilirubinemia.

Simple radiography can show gas in soft tissue, but its absence cannot dismiss NF. Thus, because gas is usually developed and detected late in the course of the disease, it is not helpful to make an initial diagnosis based on gas detection in the soft tissue. The use of ultrasound is limited to the surface structures involved or the presence of a collection of fluid or pus.^6^ Although MRI is better to diagnose soft tissue pathological conditions, the best imaging method for detecting necrosis is a CT scan. The computed tomography results show include an asymmetric thickening of the fascial associated with fat stranding and tracking of subcutaneous gases along the fascial planes. It is also helpful in evaluating the treatment outcomes because it can determine the extent of tissue involvement.^4^

A possible treatment for NF is removing the infected tissues. On the other hand, antibiotic therapy is considered a conservative treatment plan. This method aims to treat the inflammation and reduce the risk of abscess formation, allowing the inflamed bowel to heal over time.^8^ Antibiotic therapy should be started immediately and even before the test results are prepared. Nevertheless, antibiotic therapy's success depends on various factors, such as proper local blood flow and drug distribution, as if a local vascular thrombosis occurs, the release of antibiotics into the target tissues may be reduced. The recommended antibiotic therapy regimen includes β‐lactams and imidazole ±aminoglycosides. The average course of antibiotic therapy for NF is usually 4–6 weeks.^16^

The medical procedure for the departure of the reference area is called an appendectomy. Appendectomy can be performed through an open or laparoscopic medical procedure, and it is the most effective way to prevent complications is to remove the inflamed appendix early by open surgery or laparoscopically.^12^ For acute appendicitis, laparoscopic appendectomy is associated with a reduction in postoperative recovery time (*p* ˂ .025). For the north of a century, laparotomy (open appendectomy) was the standard treatment for intense a ruptured appendix. This method comprises of the expulsion of the contaminated index through a solitary enormous entry point in the lower right space of the mid‐region.^17^Some studies have shown that wound infection is less likely to occur in laparoscopic appendectomy than open surgery.^18^, ^19^, ^20^

## 4 CONCLUSIONS

We reported a rare NF case caused by perforated appendicitis, which may cause a higher mortality rate. When managing a patient with acute appendicitis, critical criteria such as good history taking and physical examination, early detection, broad‐spectrum antibiotic treatment, and proper debridement must be considered. Although acute appendicitis is one of the most common diseases in clinical practice, unusual complications such as NF should be considered, which can have fatal consequences. MRI is one of the best diagnostic methods along with CT scans, which also show the focus of intra‐abdominal infection.

## CONFLICT OF INTEREST

The authors have no competing interests in preparing and submitting this manuscript.

## AUTHOR CONTRIBUTIONS

Mohsen Rakhsha involved in conceptualization and data curation. Rezvan Hosseinzadeh involved in project administration, investigation, writing—original draft, review, and editing. Dariush Hosseinzadeh involved in writing—original draft, review, and editing. Morteza Behnamfar involved in investigation, writing—original draft. Kataneh Kazemi involved in conceptualization and investigation.

## CONSENT

Written informed consent was obtained from the patient to publish the case details and accompanying images.

## DECLARATION

This case report has never been presented, published in whole or in part.

## Data Availability

All data are included.
